# Changes of physicochemical, bioactive compounds and antioxidant activity of nutritional beverages products from VD20 broken Rice in the pasteurization process

**DOI:** 10.1016/j.fochx.2024.102018

**Published:** 2024-11-19

**Authors:** Van Tan Pham, Thi Kim Loan Le, Duc Ngoc Vu, Thi Yen Nhi Tran, Long Giang Bach, Tan Phat Dao

**Affiliations:** a Faculty of Chemical Engineering And Food Technology, Nong Lam University, Ho Chi Minh City 700000, Viet Nam; b Faculty of Agriculture and Food Technology, Tien Giang University, My Tho City, Tien Giang Province, Viet Nam; c Institute of Applied Technology and Sustainable Development, Nguyen Tat Thanh University, Ho Chi Minh City 700000, Viet Nam; d Department Chemistry, Soongsil University, Seoul 06978, South Korea

**Keywords:** VD20 broken rice, Nutrition beverage, Physicochemical properties, Pasteurization process

## Abstract

VD20 broken rice, a byproduct of rice milling, shows significant potential for value-added applications, particularly in functional beverage production. This study investigates the impact of homogenization and pasteurization on the quality and stability of VD20 broken rice-based beverages. Homogenization at speeds 6000 to 12,000 rpm for 5 to 20 min improved product stability, while pasteurization between 80 and 95 °C for up to 20 min ensured microbial safety and extended shelf life. Optimal processing conditions were achieved with homogenization at 10,000 rpm for 15 min and pasteurization at 90 °C for 15 min, maintaining a microbial count below 10^2^ CFU/mL to meet health standards. Bioactive compounds were effectively preserved, with total flavonoid content at 47.19 ± 5.30 mgQE/mL, total phenolic content at 177.12 ± 0.39 mg GAE/mL, and antioxidant activities of DPPH and ABTS at 90.87 ± 1.12 mg AAE/mL and 50.42 ± 2.05 mg AAE/mL, respectively. The final product exhibited a viscosity of 4.00 ± 0.06 cP and color values of L*: 68.21 ± 0.06, a*: 3.08 ± 0.06, b*: −2.96 ± 0.15, and ΔE: 3.21 ± 0.06. These results offer an optimized process for producing a high-quality, nutritious beverage from VD20 broken rice, enhancing its economic potential and product diversity.

## Highlights

• Homogenization at 10.000 rpm for 15 min optimizes product stability.
• Pasteurization at 90 °C for 15 min ensures microbiological safety.
• High retention of total phenolics, flavonoids, and antioxidant activities.
• Achieved desirable viscosity and color stability, ensuring product quality.

## 1 Introduction

Rice, a globally vital staple, consists of two main species *Oryza sativa* (Asian rice) and *Oryza glaberrima* (African rice), both from the Poaceae family (Loko et al., 2021). *Oryza sativa* is particularly valued for its adaptability cross diverse ecosystems and its critical role in feeding over half the world's population (The Emergence of Agriculture 0716750554, 2021). In Vietnam, the Red River Delta and the Mekong Delta represent the regions with the largest cultivated areas. Rice varieties vary in colors, including white, brown, and crimson, with carbohydrates making up 70–80 % of the grain's composition (Carcea, 2021; Rathna Priya, Eliazer Nelson, Ravichandran, & Antony, 2019). A typical 100 g serving of rice provides approximately 344 kcal, with major nutritional components such as 75.9 g of carbohydrates and 7.9 g of protein. Additionally, 100 g of rice contains important nutrients including 1.342 mg of Vitamin B5, 0.1 mg of Vitamin B1, 0.03 mg of Vitamin B2, 621 mg of aspartic acid, 546 mg of leucine, and 239 mg of lysine (Padma, Edukondalu, & Babu, 2019).

Broken rice, a byproduct of the milling, sieving, and drying processes, consists predominantly of the rice grain embryo, which is the most fragile part and rich in protein (Bee Ling Tan, 2020). Despite having a higher protein content than whole grain rice, broken rice is often undervalued and sells at lower market prices. Although it has an irregular appearance, broken rice retains its nutrients, including low-fat content, vitamins, and minerals like manganese and phosphorus. Nutritionally, broken rice is nearly identical to whole grain rice, making it a viable ingredient for various food products without significant nutrient loss. Beverages made from broken rice are beneficial for heart health, offering high carbohydrate content, omega fatty acids, vitamins, minerals, and cholesterol-free. (Arya, Salve, & Chauhan, 2016). Additionally, rice pigments contain compounds that inhibit atherosclerotic plaque formation, contributing to cardiovascular health (Z. Chen et al., 2019; Patel & Akhtar, 2018). The VD20 rice variety is a short-term fragrant rice that matures in 100–115 days and can be cultivated multiple times annually. It is known for its pests and diseases resistance and can thrive in silt-rich mangrove soils (Le et al., 2024). VD20 rice from Go Cong, Vietnam, is produced organically under European standards and is recognized for its small, milky grains and natural aroma.

In the beverage processing industry, pasteurization is a commonly employed method to eliminate harmful microorganisms and deactivate enzymes, thereby reducing risks from bacteria, viruses, yeast, and molds (Shaik & Chakraborty, 2022). This process not only ensures food safety but also extends product shelf life. Previous studies on rice-based beverages highlight the potential of rice as a versatile ingredient. For instance, (Derosa et al., 2018) developed a fermented red rice beverage combined with curcumin, which supports cardiovascular health by improving endothelial damage in individuals with hyperlipidemia. Another study by (Karimidastjerd & Kilic-Akyilmaz, 2021) enriched rice-based beverages with caseinomacropeptide to enhance bioactivity while maintaining a sensory profile like that of skim milk. (Tú, Nga, Hà, & Duy, 2016) developed a germ rice milk product using α-amylase and glucoamylase enzymes, achieving high chemical efficiency and desirable physicochemical properties, with a total phenolic content of 111.03 ± 0.5 mg GAE/mL and a viscosity of 3.37 cP. Another study by (Plengsaengsri et al., 2019) focused on developing a milk substitute suitable for individuals with allergies to animal proteins and lactose, yielding a rice milk product with a comparable nutritional profile to cow's milk.

These studies underscore the growing interest in rice-based products and the importance of optimizing processing techniques to enhance their nutritional value and safety (Wang et al., 2022). However, research on utilizing rice milling by products, such as broken rice, remains limited. Despite its potential, VD20 broken rice has not been extensively studied for beverage applications, facing challenges like short shelf life and susceptibility to microbial spoilage. Addressing these issues through pasteurization is promising, as thermal pasteurization is scalable and effective, though it may impact physicochemical properties and nutritional quality.

For these reasons, this study investigates the underutilized potential of VD20 broken rice, a nutritious byproduct often undervalued in the market. By converting VD20 broken rice into a stable, nutritious beverage, this research aims to diversify products and increase the economic value of the VD20 variety, benefiting agricultural regions such as Go Cong in Tien Giang province. This study employs two main steps: refining processing techniques to enhance beverage stability and ensuring safety and quality through physicochemical and bioactive assessments after pasteurization to retain quality and prolong shelf-life. The resulting VD20 broken rice beverage aligns with functional food trends, offering a high-quality, antioxidant-rich product supporting cardiovascular health and specific dietary needs, thus contributing to both food industry innovation and sustainable agriculture.

## 2 Experimental

### 2.1 Materials

VD20 broken rice was sourced from HK Green Production and Production Co., Ltd., My Tho City, Tien Giang province, Vietnam. Enzymes α-amylase (100,000 U/mL) and glucoamylase (250,000 U/mL) were purchased from AngelYeast Co., Ltd., China, along with other reagents such as Folin-Ciocalteu, quercetin standard, and DPPH. Additional chemicals, including AlCl₃ solution (10 %), Na₂CO₃ 20 %, peptone, NaCl, KH₂PO₄, Na₂HPO₄·12H₂O were sourced from China and CH₃COOK solution (1 M) was sourced from Vietnam.

### 2.2 Processes of production nutritional beverages from VD20 broken rice

The production process for the VD20 broken rice beverage includes serval key steps. Initially, the rice was thoroughly washed to remove impurities. The cleaned rice was then roasted at 200 °C for 15 min to enhance the flavor profile of the final product. After roasting, the rice was finely ground and gelatinized in water at 95 °C for 15 min.

The gelatinized rice solution was then subjected to enzymatic hydrolysis in two stages: liquefaction and saccharification. During liquefaction, α-amylase (100 U/g) was added to break down long carbohydrate chains, followed by glucoamylase (200 U/g) in the saccharification stage to convert short-chain sugars into simple sugars (R. Chen, Li, Liu, Yang, & Li, 2012; Tong, Tong, & Shi, 2019). After filtration, stabilizers and crushed cashew nut fluid were added to the solution incorporated into the solution. The final mixture was then homogenized, pasteurized, and bottled.

#### 2.2.1 Experimental setup and analysis

To evaluate the stability of the produced beverage, experiments were conducted on 16 samples using KA T50 digital ULTRA-TURRAX homogenizer at different rotational speeds (6000, 8000, 10,000, and 12,000 rpm) and time intervals (5, 10, 15, and 20 min). After determining the optimal homogenization parameters, further tests were performed to assess the effects of pasteurization time and temperature, with samples treated at four temperatures (80, 85, 90, and 95 °C) and four-time intervals (5, 10, 15, and 20 min). Parameters analyzed included stability, color, viscosity, total polyphenol content, total flavonoid content, DPPH antioxidant activity, ABTS scavenging activity, and total aerobic microorganism count.

### 2.3 Determination of viscosity and color analysis

The viscosity of the product was measured using a Brookfield viscometer (ViscoQc 100 L, US) at 100 rpm. Color was evaluated with a Konica Minolta (CR400, Japan) handheld colorimeter, using the the CIELab color system, where L* represents lightness, a* denotes the red-green axis, and b* represents the yellow-blue axis.

The total color difference of the samples before and after the pasteurization process was calculated using Eq. 1:

(1) $$∆E=\sqrt{{\left(L1-L2\right)}^{2}+{\left(a1-a2\right)}^{2}+{\left(b1-b2\right)}^{2}\;}$$

where ∆E is total color difference, and L1, a1, b1, are the values before pasteurization, while L2, a2, b2 are the values after pasteurization.

### 2.4 Determination of total polyphenol content

TPC was quantified using the Folin-Ciocalteu method, with gallic acid served as the standard (Bui T. Thu Thao et al., 2023; Vu et al., 2022). A 10 mL beverage sample was diluted to 50 mL with alcohol, then 0.1 mL of this solution was mixed with 0.5 mL of 10 % Folin-Ciocalteu reagent, and 0.4 mL of 7.5 % Na₂CO₃ solution, and allowed to react in the dark at room temperature for 1 h. Absorbance was measured at 765 nm using a UV–Vis spectrophotometer, with TPC calculated using Eq. 2:

(2) $$\mathrm{TPC}=\frac{C\times V\times \mathrm{df}}{V_{\mathrm{sample}}\times 1000}$$

where C is the TPC concentration from the calibration curve (μg/mL), V is volume of extracted fluid (mL), df: dilution coefficient, and V _sample_ is the sample volume (mL), 100/1000: conversion factor from μg/mL to mg/100 mL.

### 2.5 Determination of total flavonoid content

TFC was measured by the AlCl_3_ colorimetric method by (Do et al., 2024; Bui Thi Thu Thao et al., 2024) with quercetin as the standard. A 10 mL sample was diluted to 50 mL with alcohol, followed by the addition of 0.5 mL of the extracted solution, 4.3 mL of alcohol, 0.1 mL of 10 % AlCl₃ solution, and 0.1 mL of 1 M CH₃COOK solution. After shaking, the mixture reacted at room temperature for 30 min, and the absorbance was recorded at 415 nm. TFC was calculated using Eq. 3:

(3) $$\mathrm{TFC}=\frac{C\times V\times \mathrm{df}}{V_{\mathrm{sample}}\times 1000}$$

### 2.6 Determination of antioxidant activity

#### 2.6.1 DPPH scavenging activity

Antioxidant activity was evaluated using the DPPH free radical scavenging method (Linh et al., 2024; Tran et al., 2023). A 10 mL sample was diluted to 50 mL with alcohol. The reaction mixture, containing 0.5 mL of sample and 1.5 mL of standard ABTS, was incubated in the dark at room temperature for 30 min. Absorbance was recorded at 517 nm, and DPPH activity was calculated with Eq. 4:

(4) $$\text{DPPH}=\frac{A_{1}-A_{2}}{A_{1}}\times 100$$

where A_1_ is the absorbance of the blank, and A_2_ is the absorbance of the sample after 30 min.

**b. ABTS Radical Scavenging Activity.**

ABTS radical scavenging was assessed similarly (Dao, Vu, Nguyen, Pham, & Tran, 2022; Nhi Yen Thi Tran, Bach, & Huynh, 2022). A 10 mL sample was diluted to 50 mL with alcohol, followed by the addition of 0.5 mL sample and 1.5 mL standard ABTS. The reaction proceeded in the dark for 30 min, with absorbance measured at 734 nm. ABTS activity was calculated using Eq. 5:

(5) $$\text{ABTS}=\frac{A_{1}-A_{2}}{A_{1}}\times 100$$

### 2.7 Microbiological indicators

The total number of aerobic microorganisms was quantified using the agar plate count method on plate count agar medium (Le et al., 2024; Nhi et al., 2022). Samples were incubated aerobically at 30 °C or 35 °C for 24–48 h. The microorganism count per mL was calculated based on colony numbers, with the permissible limit set at 102 CFU/mL (TCVN 6507–1:2005, TCVN 6404:2008, TCVN 4884–1:2015).

### 2.8 Stability determination methods

Stability was assessed by measuring the separation layer's height relative to the total fluid volume after storage under cooler conditions for 24 h, following the method by (Tan, Ng, Tan, Chong, & ⁎Tan, C.P., 2019). A 10 mL sample of the product was stored under cooler conditions, and the height of the separate layer was recorded after 24 h. The stability index was calculated using Eq. 6:

(6) $$\mathrm{SI}\;\left(\%\right)=\left[\frac{H_{1}}{H_{0}}\right]*100$$

where H_1_ is the height of the separated layer and H_0_ is the total height of the liquid.

### 2.9 Data processing methods

All experiments were conducted in triplicate, with results presented as mean ± standard deviation. Data analysis was conducted using two-way ANOVA and LSD tests, processed with Statgraphics Centurion XV 9 software (Statgraphics Technologies, Inc., USA). Statistical significance was set at a *p*-value of <0.05, with a 95 % confidence interval applied to all analyses.

## 3 Result and discussions

### 3.1 Effects of time and rate of homogenization on drinking water stability of VD20 broken rice

The stability and uniformity of the emulsion are essential indicators of product quality, with phase separation percentage serving as a key metric for stability. Lower phase separation corresponds to smaller dispersed particles, yielding a more stable emulsion (Sidhu & Singh, 2016).

**Fig. 1:**
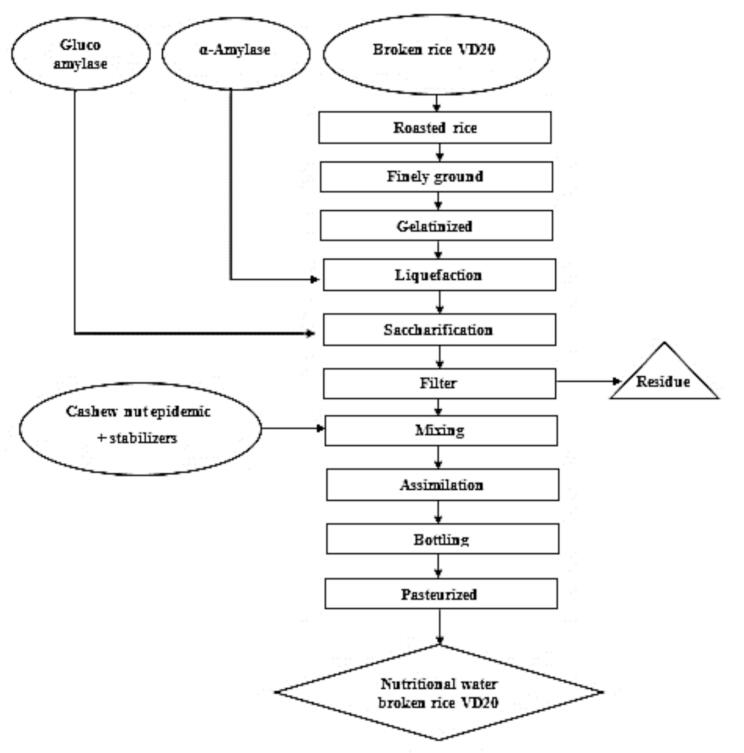
Production process of nutritional drink broken rice VD20. where (a-d) indicates a statistically significant difference (p < 0.05).

Table 1 demonstrates the impact of homogenization on phase separation. Notably, samples homogenized at 6000 rpm exhibited higher phase separation compared to those homogenized at 8000, 10,000, and 12,000 rpm. Specifically, at 6000 rpm, phase separation decreased from 5.66 % to 4.65 % as homogenization time increased from 5 to 20 min. This pattern aligns with findings from studies on milk and beverages, where homogenization reduces fat globule size, promoting a stable emulsion (Ningtyas, Bhandari, & Prakash, 2021). However, at 12,000 rpm, phase separation percentages varied slightly—5.09 %, 4.69 %, 4.85 %, and 5.13 % over 5–20 min—indicating that homogenization speed significantly influences stability. While higher speeds cause more extensive fragmentation of fat particles, leading to even dispersion in the emulsion (Fernandez-Avila & Trujillo, 2016). The intense rotational force at 12,000 rpm can induce rapid phase separation, reducing stability compared to outcomes observed at 8000 and 10,000 rpm. The lowest phase separation, 3.31 %, occurred at 10,000 rpm for 15 min, indicating optimal homogenization conditions for maximum emulsion stability. (See Fig. 1.)

**Table 1:** The result affects the homogenization process on the stability of nutritious drinking water of broken rice VD20 after 24 h (%).

Time (min)	Homogenization rate (rpm)			
	6.000	8.000	10.000	12.000
5	5.66 ± 0.19^a1^	5.16 ± 0.77^a2^	4.80 ± 0.79^a2^	5.09 ± 0.75^a1^
10	4.79 ± 0.15^cb1^	4.43 ± 0.4^cb2^	4.23 ± 0.06^cb2^	4.69 ± 0.75^cb1^
15	4.69 ± 0.05^c1^	4.27 ± 0.03^c2^	3.31 ± 0.79^c2^	4.85 ± 0.70^c1^
20	4.65 ± 0.79^b1^	4.67 ± 0.77^b2^	4.25 ± 0.03^b2^	5.13 ± 0.73^b1^

a1, a2, b1, b2, c1, c2, cb1, cb2:Values can show Statistically significant difference at p < 0.05.

ANOVA and LSD tests confirmed significant differences among homogenization conditions, with statistically significant variations (*P* < 0.05) as time and speed increased. A slight rise in phase separation was noted at 15 min for 12,000 rpm and 20 min across other speeds, likely due to exceeding the dispersion limit, which leads to fat droplet reaggregation (Tangsuphoom & Coupland, 2008). In summary, homogenization effectively enhances product stability by reducing phase separation. This study identifies 15 min at 10,000 rpm as the optimal homogenization condition for achieving maximum product stability.

### 3.2 Effects of pasteurization on the quality of nutritious beverage made from VD20 broken Rice

#### 3.2.1 Effects of pasteurization on microbial stability

Pasteurization is widely used to inhibit microbial growth and enzyme activity in food product (Yousef & Balasubramaniam, 2012). According to Vietnamese Ministry of Health regulations (46/BYT and Circular No.35/2010/TT-BYT), the permissible limit for total aerobic microorganisms in non-alcoholic beverages is <10^2^ CFU/mL.

Table 2 displays the effects of pasteurization temperature and time on microbial counts in the VD20 broken rice beverage. At 80 °C, microbial counts remained relatively high but decreased as temperature and duration increased, indicating that lower temperatures provide insufficient heat energy for effective microbial inactivation. For example, at 85 °C, microbial counts were recorded as 8.8 × 10^2^, 7.5 × 10^2^, 3.7 × 10^2^, and 1.9 × 10^2^ CFU/mL for pasteurization times of 5, 10, 15, and 20 min, respectively. This inverse relationship between pasteurization time and microbial density highlights the importance of extended pasteurization for effective microbial control (Timmermans et al., 2011). Increasing the pasteurization temperature to 90 °C for 10 min resulted in a microbial density of 0.6 × 10^2^ CFU/mL, adhering to the safety standards established by Decision No. 46/2007/QD-BYT and Circular No. 35/2010/TT-BYT. This demonstrates the critical role of temperature in reducing microbial counts. Higher temperatures, in the range of 85–95 °C, are more effective in killing microorganisms, enhancing product safety and quality. Notably, at 90 °C for 15–20 min and 95 °C for 5–20 min, no aerobic microorganisms were detected (Silva & Gibbs, 2004).

**Table 2:** The result of a transformation of total aerobic microorganisms with temperature and pasteurization time (CFU/mL).

Time (min)	Pasteurization temperature (°C)			
	80	85	90	95
5	1.5 × 10^3^	8.8 × 10^2^	1.3 × 10^2^	–
10	9.4 × 10^2^	7.5 × 10^2^	0.6 × 10^2^	–
15	6.1 × 10^2^	3.7 × 10^2^	–	–
20	3.0 × 10^2^	1.9 × 10^2^	–	–

-: no detection. Results were expressed as less than 10 CFU/g or less than 1 CFU/mL, respectively, “not detected” when no colonies grew on the plate.

The results indicate that increasing pasteurization temperatures significantly reduce microbial counts in VD20 broken rice nutritious drinking water, with effective microbial inactivation achieved at higher temperatures. Pasteurization at 80–85 °C was found to be inadequate, as it resulted in high microbial densities and reduced safety. The optimal pasteurization conditions were determined to be 90 °C for 10–15 min and 95 °C for 5–20 min, where microbial levels remained within acceptable limits. However, high-temperature pasteurization may negatively impact product quality and nutritional value, affecting phenolic compounds, flavonoids, and antioxidants (Ravichandran, Jayachandran, Kothakota, Pandiselvam, & Balasubramaniam, 2023). Balancing pasteurization parameters is crucial to ensure product safety while minimizing quality degradation. Further evaluation is needed to refine pasteurization conditions to maintain both safety and nutritional integrity.

#### 3.2.2 The effect of temperature and pasteurization time on color

**Fig. 2:**
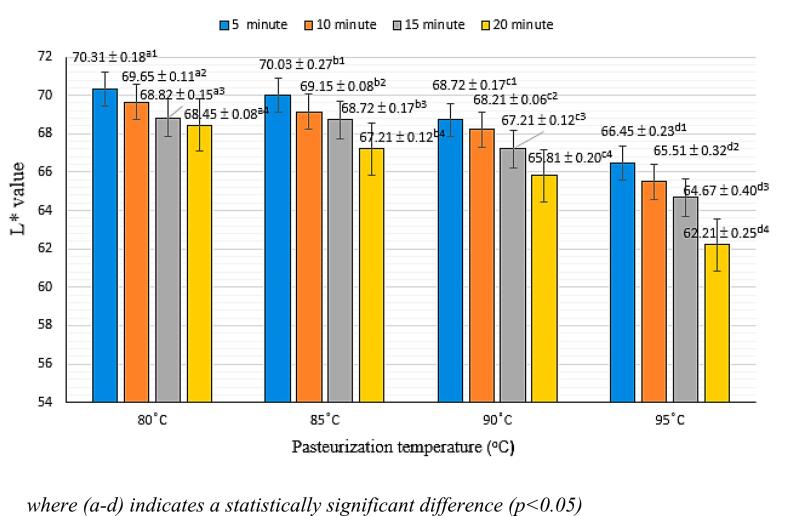
Effect of pasteurization on L*.

Pasteurization not only extends shelf life by inhibiting microbial growth but also impacts the color of VD20 broken rice drink. Fig. 2 demonstrates that both pasteurization time and temperature influence the product's color.

The L* value, indicating lightness, decreases with increasing pasteurization temperature and duration. Similar results were observed in fruit, color degradation occurred with prolonged heat retention at 90 °C (Sonar, Rasco, Tang, & Sablani, 2019). Pre-pasteurization, the L* value of the product was 72.19. Following pasteurization at 80 °C, the L* value decreased progressively from 70.31 to 68.45 over 5–20 min, indicating a 2.64 % reduction. The most significant color change was observed at 95 °C, where the L* value dropped to 62.21 after 20 min due to the Maillard reaction, in which sugars and amino acids interact at high temperatures (Dewanto, Wu, & Liu, 2002). ANOVA analysis revealed a significant effect of temperature on L* value at 95 °C (*p* < 0.05).

Table 3 further illustrates the ΔE, which increases with higher pasteurization temperatures and longer durations, particularly at 95 °C. The statistical significance of this deviation (p < 0.05) emphasizes the impact of pasteurization on product color. Indeed, higher pasteurization temperatures and extended times accelerate browning, affecting product color (Wibowo et al., 2019). Optimal color retention was observed with pasteurization at 90 °C for 5–10 min, balancing microbial safety with minimal color degradation.

**Table 3:** The result of the effect of pasteurization mode on color deviation *∆E.*

Time (min)	Pasteurization temperature (°C)			
	80	85	90	95
5	1.26 ± 0.05^c3^	1.49 ± 0.06^c3^	2.70 ± 0.17^b3^	4.92 ± 0.03^a3^
10	1.83 ± 0.04^c32^	2.29 ± 0.011^c32^	3.21 ± 0.06^b32^	5.91 ± 0.02^a32^
15	2.62 ± 0.02^c21^	2.70 ± 0.17^c21^	4.19 ± 0.04^b21^	6.69 ± 0.40^a21^
20	2.97 ± 0.02^c1^	4.18 ± 0.12^c1^	5.58 ± 0.13^b1^	9.15 ± 0.02^a1^

a1, a21, a3, a32, b1, b21, b3, b32, c1, c2, c3, c32:Values can show Statistically significant difference at p < 0.05.

#### 3.2.3 The effect of temperature and pasteurization time on product viscosity

**Fig. 3:**
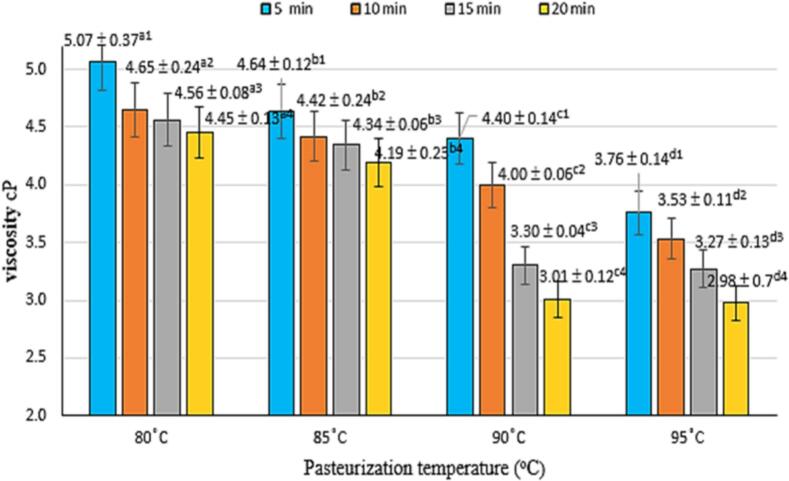
Effect of pasteurization time and temperature on product viscosity. * Where (a-d) and (1–4) indicate a statistically significant difference (p < 0.05).

Pasteurization temperature and time also influence the viscosity of VD20 broken rice drink. As shown in Fig. 3, viscosity decreases with increasing temperature and prolonged heat retention. The highest viscosity of 5.07 cP was observed at 80 °C for 5 min, while the lowest viscosity of 2.98 cP occurred at 95 °C for 20 min.

Typically, higher temperatures cause water loss in products, increasing viscosity, as seen in tamarind sauce and roasted products (Witono, Windrati, Taruna, Afriliana, & Assadam, 2014). However, in this case, the decrease in viscosity with higher temperatures can be explained by the denaturation of proteins, which serve as protective agents for fat droplets in the emulsion. At high temperatures, these proteins lose their protective capacity, leading to a reduction in viscosity (Mohammadian & Madadlou, 2018). ANOVA and LSD tests indicated significant effects of temperature and time on viscosity (p < 0.05). An optimal viscosity range was observed at 85–90 °C for 5–15 min, balancing texture and stability.

#### 3.2.4 Effect of temperature and pasteurization time on total polyphenol content

Table 4 reveals that TPC initially increased with pasteurization time and temperature but declined to higher levels. Polyphenols are sensitive to temperature, and their content can be influenced by thermal processing (Cao et al., 2021).

**Table 4:** TPC content of broken rice nutritional drink VD20.⁎

Time (min)	Pasteurization temperature (°C)			
	80	85	90	95
5	103.73 ± 0.77^d21^	114.89 ± 1.69^c21^	166.03 ± 1.12^a21^	164.76 ± 0.18^b21^
10	105.63 ± 1.07^d1^	129.14 ± 0.62^c1^	177.12 ± 0.39^a1^	149.92 ± 0.05^b1^
15	110.95 ± 0.17^d2^	136.99 ± 0.55^c2^	170.83 ± 1.18^a2^	123.56 ± 0.13^b2^
20	114.87 ± 1.74^d3^	133.16 ± 1.40^c3^	168.44 ± 2.91^a3^	99.09 ± 0.94^b3^

a1, a2, a21, a3, b1, b2, b21, b3, c1, c2, c21, c3, d1, d2, d21, d3:Values can show Statistically significant difference at p < 0.05.

⁎ Unit: (mgGAE/mL).

At 90 °C, TPC peaked at 177.12 mgGAE/mL after 10 min, decreasing slightly to 170.83 mgGAE/mL at 15 min, and further at 95 °C to 149.92 mgGAE/mL after 10 min. Moderate heat enhances polyphenol release, while excessive heat leads to degradation. (Ioannou & Ghoul, 2012). ANOVA analysis indicates significant differences in TPC across different pasteurization conditions, with the optimal TPC achieved at 90 °C for 10 min.

#### 3.2.5 The effect of temperature and pasteurization time on the total flavonoid content

The data presented in Table 5 indicate that the TFC generally increased with temperature and time up to an optimal level. Beyond this threshold, extended heat retention and higher pasteurization temperatures led to a deterioration in TFC content. Specifically, at a pasteurization temperature of 80 °C, the TFC values increased with time, reaching a peak of 48.67 ± 2.03 mgQE/mL in 15 min, before slightly decreasing to 46.87 ± 2.01 mgQE/mL at 20 min. Similarly, at 85 °C, TFC values peaked at 55.27 ± 2.37 mgQE/mL at 15 min, with a slight decrease to 49.20 ± 3.87 mgQE/mL at 20 min.

**Table 5:** TFC content of broken rice nutritional drink VD20 (mgQE/mL).⁎

Time (min)	Pasteurization temperature (°C)			
	80	85	90	95
5	31.53 ± 1.08^c2^	40.43 ± 3.38^a2^	46.98 ± 5.14^b2^	45.02 ± 3.83^d2^
10	33.38 ± 0.20^c2^	48.08 ± 3.47^a2^	47.19 ± 5.30^b2^	38.79 ± 4.09^d2^
15	48.67 ± 2.03^c1^	55.27 ± 2.37^a1^	45.90 ± 2.98^b1^	31.74 ± 2.48^d1^
20	46.87 ± 2.01^c2^	49.20 ± 3.87^a2^	39.42 ± 2.00^b2^	25.18 ± 2.28^d2^

a1, a2, b1, b2, c1, c2, d1, d2:Values can show Statistically significant difference at p < 0.05.

⁎ Unit: (mgQE/mL).

The highest TFC content of 55.27 ± 2.37 mgQE/mL was recorded at 85 °C for 15 min, which was significantly higher compared to other conditions. However, beyond this optimal point, TFC content decreased sharply. At 95 °C in 20 min, the TFC content fell to 25.18 ± 2.28 mgQE/mL. ANOVA and LSD analysis indicated significant differences in TFC content across different temperatures and times, with *p*-values <0.05 and a 95 % confidence level (a-d). The results suggest that the optimal pasteurization conditions for maintaining high TFC content are 85 °C for 15 min. This condition maximizes TFC content while minimizing degradation.

#### 3.2.6 The effect of temperature and pasteurization time on antioxidant activity (DPPH, ABTS)

Antioxidant capacity, as measured by DPPH and ABTS assays, is a critical parameter for evaluating the health benefits of food products. The results presented in Table 6 and Table 7 demonstrate that the antioxidant activity of broken rice water showed varying trends with changes in pasteurization temperature and time.

**Table 6:** DPPH antioxidant activity of VD20 broken rice nutrition drink.⁎

Time (min)	Pasteurization temperature (°C)			
	80	85	90	95
5	62.88 ± 2.47^c32^	66.93 ± 2.72^b32^	83.42 ± 4.01^a32^	82.36 ± 1.93^b32^
10	65.05 ± 3.61^c1^	70.27 ± 2.68^b1^	90.87 ± 1.12^a1^	77.34 ± 1.83^b1^
15	68.75 ± 3.95^c21^	75.54 ± 2.54^b21^	86.87 ± 3.10^a21^	68.22 ± 1.78^b21^
20	70.58 ± 1.01^c3^	73.71 ± 2.50^b3^	83.78 ± 3.13^a3^	60.79 ± 2.40^b3^

a1, a21, a3, a32, b1, b21, b3, b32, c1, c21, c3, c32:Values can show Statistically significant difference at p < 0.05.

⁎ Unit: mgAAE/mL.

**Table 7:** ABTS antioxidant activity of VD20 broken rice nutrition drink.⁎

Time (min)	Pasteurization temperature (°C)			
	80	85	90	95
5	28.28 ± 1.94^c1^	32.15 ± 2.25^b1^	50.37 ± 2.45^a1^	46.70 ± 1.03^b1^
10	30.45 ± 2.03^c1^	35.48 ± 2.19^b1^	50.42 ± 2.05^a1^	43.06 ± 1.18^b1^
15	34.40 ± 4.69^c1^	41.04 ± 2.72^b1^	51.01 ± 1.65^a1^	32.81 ± 5.02^b1^
20	38.27 ± 0.39^c2^	38.63 ± 3.29^b2^	48.59 ± 2.46^a2^	25.57 ± 3.01^b2^

a1, b1, c1:Values can show Statistically significant differences at p < 0.05.

⁎ Unit: mgAAE/mL.

Table 6 shows that DPPH radical scavenging activity increased with pasteurization time at 80 °C, rising by 10.9 % from 5 to 20 min. A similar increase was observed at 85 °C, with an 11.4 % rise from 5 to 15 min. However, further extension of the pasteurization time at these temperatures did not significantly enhance antioxidant activity. DPPH radical scavenging activity peaked at 90 °C for 10 min but declined at longer times and higher temperatures (90 °C and 95 °C), indicating that extended heat exposure can lead to a reduction in antioxidant capacity. This decline is likely due to the degradation of polyphenols and other antioxidant compounds under high temperatures (Zeng, Ma, Li, & Luo, 2017).

Table 7 highlights that ABTS activity was highest at 90 °C, with a value of 51.01 ± 1.65 mgAAE/mL. The antioxidant activity measured by ABTS was more stable at 90 °C compared to other temperatures. At 95 °C, a significant decrease in ABTS activity was observed, highlighting that higher temperatures can adversely affect antioxidant activity. LSD grading tests indicated no significant difference in antioxidant activity between pasteurization times of 5–15 min, but a notable difference was found at 20 min. ANOVA analysis revealed a statistically significant difference at 95 °C (*p* < 0.05), suggesting that high temperatures can cause the evaporation or degradation of antioxidant compounds, reducing their biological activity. Optimal antioxidant retention was found at 90 °C for 15 min for ABTS activity and 90 °C for 10 min for DPPH activity.

#### 3.2.7 The effect of temperature and pasteurization time on product stability

**Fig. 4:**
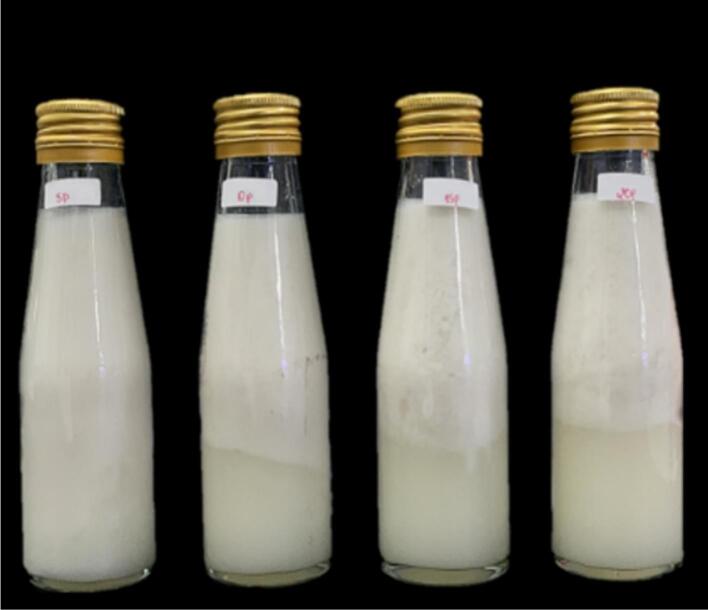
Sample of nutritious drinking water for broken rice VD20, pasteurized 95 °C.

Increasing the pasteurization temperature to 95 °C and extending the heat retention time to 5, 10, 15, and 20 min significantly affected the physical state of the product, as compared to lower temperatures and shorter pasteurization times of 80 °C, 85 °C, and 90 °C. These effects are visually demonstrated in Fig. 4.

Fig. 4 illustrates the physical changes in the product subjected to pasteurization at 95 °C for varying times (5 to 20 min). The product showed increasing amounts of visible precipitate as pasteurization time increased. Specifically, the sample pasteurized for 5 min exhibited the least phase separation, while the samples processed for longer times displayed more pronounced layering and phase differentiation. The most significant effect was observed in the sample pasteurized for 20 min, which had a substantial amount of precipitate and a noticeable loss of product clarity. Shivani et al. showed that at high temperatures, such as those used in pasteurization, proteins tend to coagulate, resulting in a change in product texture and appearance (Pathania, Parmar, & Tiwari, 2019). In the case of VD20 broken rice nutritious drinking water, the maximum pasteurization temperature of 95 °C led to the formation of a cotton-like precipitate and thin whey, indicative of protein coagulation and a reduction in the product's cohesive properties.

Moreover, the VD20 broken rice beverage contains cashew nut crush, which has been reported to experience protein denaturation and coagulation at high temperatures. Studies on cashew milk have shown that protein denaturation occurs around 90–95 °C, depending on the product's specific characteristics (da Silva, Velasco, & Fakhouri, 2023). This denaturation contributes to the observed changes in the physical state of the product. Thus, while high-temperature pasteurization effectively kills microorganisms and enhances microbiological safety, excessive heat can lead to undesirable physical changes in the product (Dash et al., 2022). Based on the observed effects, a pasteurization temperature of 90 °C is recommended to balance effective microbial control with minimal physical alteration.

## 4 Conclusion

This study established optimal processing conditions for producing a stable, nutrient-rich beverage from VD20 broken rice. Homogenization at 10,000 rpm for 15 min, combined with pasteurization at 90 °C for 10 min, resulted in a high-quality beverage with preserved bioactive compounds and microbial safety within acceptable limits. At the same time, the content of total polyphenols, total flavonoids and antioxidant activity against free radicals DPPH and ABTS at this pasteurization mode respectively reached 177.12 ± 0.39 mgGAE/mL, 47.19 ± 5.30 mgQE/mL, DDPH was 90.87 ± 1.12 mgAAE/mL, ABTS was 50.42 ± 2.05 mgAAE/mL. Suitable physicochemical indicators such as viscosity 4.00 ± 0.06 cP, L*: 68.21 ± 0.06, a*: 3.08 ± 0.06, b*: −2.96 ± 0.15, ∆E: 3.21 ± 0.06. The values achieved at the 90 °C -10 min pasteurization mark have negligible variation. This research succeeded in contributing to perfecting the technological process of producing nutritious drinking water based on broken rice from raw materials of VD20 rice Go Cong, Tien Giang province.

## Code availability

Not applicable.

## Ethics approval

Not applicable.

## Consent to partipate

Not applicable.

## Consent for publication

Not applicable.

## CRediT authorship contribution statement

**Van Tan Pham:** Writing – original draft, Validation, Software, Investigation. **Thi Kim Loan Le:** Validation, Software, Methodology. **Duc Ngoc Vu:** Validation, Software, Methodology. **Thi Yen Nhi Tran:** Software, Methodology, Formal analysis, Conceptualization. **Long Giang Bach:** Validation, Supervision, Project administration, Conceptualization. **Tan Phat Dao:** Writing – review & editing, Writing – original draft, Validation, Supervision, Project administration, Investigation, Formal analysis.

## Declaration of competing interest

The authors declare that they have no known competing financial interests or personal relationships that could have appeared to influence the work reported in this paper.

## Data Availability

Data will be made available on request.
